# The evolution of thymic lymphomas in p53 knockout mice

**DOI:** 10.1101/gad.252148.114

**Published:** 2014-12-01

**Authors:** Crissy Dudgeon, Chang Chan, Wenfeng Kang, Yvonne Sun, Ryan Emerson, Harlan Robins, Arnold J. Levine

**Affiliations:** 1 Rutgers Cancer Institute of New Jersey, New Brunswick, New Jersey 08901, USA;; 2 Department of Medicine, Rutgers Robert Wood Johnson Medical School, New Brunswick, New Jersey 08901, USA;; 3 Biomarker Discovery, Adaptive Biotechnologies, Seattle, Washington 98102, USA;; 4 Public Health Sciences, Fred Hutchinson Cancer Research Center, Seattle, Washington 98109, USA:; 5 Simons Center for Systems Biology, School of Natural Sciences, Institute for Advanced Study, Princeton, New Jersey 08540, USA

**Keywords:** p53, T-cell lymphoma, mutation rate, clonality, Pten, chromosome abnormalities

## Abstract

Germline deletion of the p53 gene in mice gives rise to spontaneous thymic (T-cell) lymphomas. In this study, the p53 knockout mouse was employed as a model to study the mutational evolution of tumorigenesis. The clonality of the T-cell repertoire from p53 knockout and wild-type thymic cells was analyzed at various ages employing TCRβ sequencing. These data demonstrate that p53 knockout thymic lymphomas arose in an oligoclonal fashion, with tumors evolving dominant clones over time. Exon sequencing of tumor DNA revealed that all of the independently derived oligoclonal mouse tumors had a deletion in the *Pten* gene prior to the formation of the TCRβ rearrangement, produced early in development. This was followed in each independent clone of the thymic lymphoma by the amplification or overexpression of cyclin Ds and Cdk6. Alterations in the expression of Ikaros were common and blocked further development of CD-4/CD-8 T cells. While the frequency of point mutations in the genome of these lymphomas was one per megabase, there were a tremendous number of copy number variations producing the tumors’ driver mutations. The initial inherited loss of p53 functions appeared to delineate an order of genetic alterations selected for during the evolution of these thymic lymphomas.

## Abstract (precis)

Germline deletion of p53 in mice gives rise to spontaneous thymic lymphomas. Dudgeon et al. demonstrate that p53 knockout thymic lymphomas arise in an oligoclonal fashion, with tumors evolving dominant clones over time. All of the independently derived oligoclonal mouse tumors had a deletion in the Pten gene prior to the formation of the TCRβ rearrangement. This was followed by the amplification or overexpression of cyclin Ds and Cdk6. This study details the mutational evolution of thymic lymphoma tumorigenesis.

Mutations in the p53 gene arise in >50% of all human cancers (Hollstein et al. 1991; Levine et al. 1991), and individuals with germline mutations in the *TP53* gene develop Li-Fraumeni syndrome, a disorder that increases the risk of developing many different cancers, especially in children and young adults (Malkin 2011). The p53 protein functions as a transcription factor to induce cell cycle arrest, apoptosis, and senescence following genotoxic stress. It does this mainly by enhancing the transcription of an array of genes, such as *Cdkn1a* (p21) and *BBC3* (Puma), which carry out these cellular functions. Because of its abilities to protect cells from accumulating DNA damage following genotoxic stress, p53 has been described as the “guardian of the genome” (Lane 1992). Other cellular functions that p53 has been known to regulate transcriptionally include DNA repair, metabolism, autophagy, angiogenesis, and antioxidant potential (Levine and Oren 2009).

Loss of p53 expression in p53 knockout mice reveals a role for p53 in the protection of mice from spontaneous tumorigenesis. The majority of p53 knockout mice succumb to thymic CD4^+^CD8^+^ double-positive T-cell lymphomas (Donehower et al. 1995), but the events responsible for the formation and evolution of these tumors in p53 knockout mice remain poorly understood. For example, it is unclear what the mutation frequencies in these thymic lymphomas are during their development. Could these tumors arise from a single clone, or are they oligoclonal? If they are oligoclonal, do these clones compete, and will a dominant clone arise with time? What are the mutations that drive this process? Do all of the tumors that arise in different p53 knockout mice have the same driver genes altered to produce these tumors? Is the order in which these driver mutations arise important for the selection process in forming a lymphoma? All of these questions are addressed and answered in this study.

## Results

### Assessing the clonality of thymic T cells in wild-type and p53 knockout T cells and thymic lymphomas

**Figure 1.**
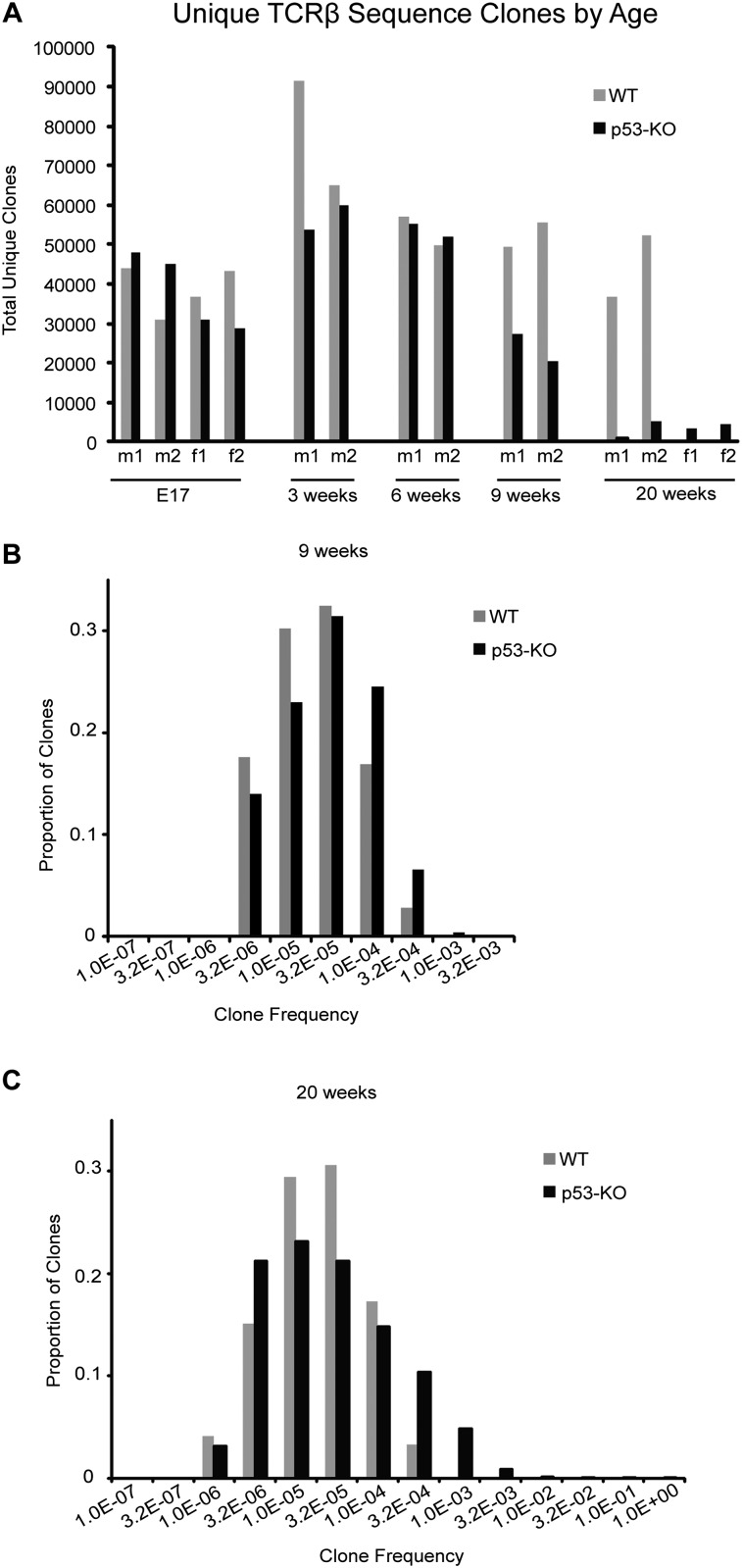
T-cell clonality decreases in p53 knockout (p53-KO) thymus. (*A*) The total number of unique clones represents the number of TCRβ sequencing reads that are different within a sample. (m) Male; (f) female. (*B*,*C*) Histograms showing distribution of clones binned by their clonal frequencies at 9 wk and 20 wk.

p53 knockout mice develop thymic lymphomas over the first 6 mo of their lives. As each T cell arises in the thymus, it is marked with a unique T-cell receptor, and so one can assess the clonal lineages of a T cell by sequencing the V-D-J region of the DNA that defines a T cell and its clonal progeny (Robins et al. 2009; Gopalakrishnan et al. 2013). The number of identical V-D-J DNA sequencing reads is proportional to the number of offspring from each T-cell clone. To establish the baseline of unique copy and replicated T-cell clones in a thymus from wild-type C57Bl/6 mice, the DNA from a thymus was extracted from two male and two female mice at 17 d of embryonic life (E17) and 3 wk, 6 wk, 9 wk, and 20 wk after birth. The PCR primers were located in the V region spanning to the constant region of the TCR-β chain DNA. The PCR products were sequenced, and the number of unique T-cell clones was determined as well as the frequency of oligoclonal T cells in the thymus (Table 1, the percentage is given for the two most common clones observed). Table 1 provides the frequency of T-cell clones in the thymus of a typical wild-type mouse. Possible PCR primer bias, which can alter the number of PCR copies that are then sequenced, was corrected by employing the algorithms provided by Adaptive Biotechnologies (Carlson et al. 2013). The highest frequency of replicated T-cell clones in the wild-type thymus varied between 0.08% and 0.17% of the total T cells sequenced (average of 0.11% ± 0.05%) (Table 1). In the thymus of the p53 knockout mice, these numbers remained the same as wild-type mice from E17 through 6 wk after birth (0.14% ± 0.054; *P* = 0.54, Student’s *t*-test). By 9 wk after birth, the number of unique clones in the thymus began to decline, and the frequency of replicated clones increased (Fig. 1).

**Table 1.**
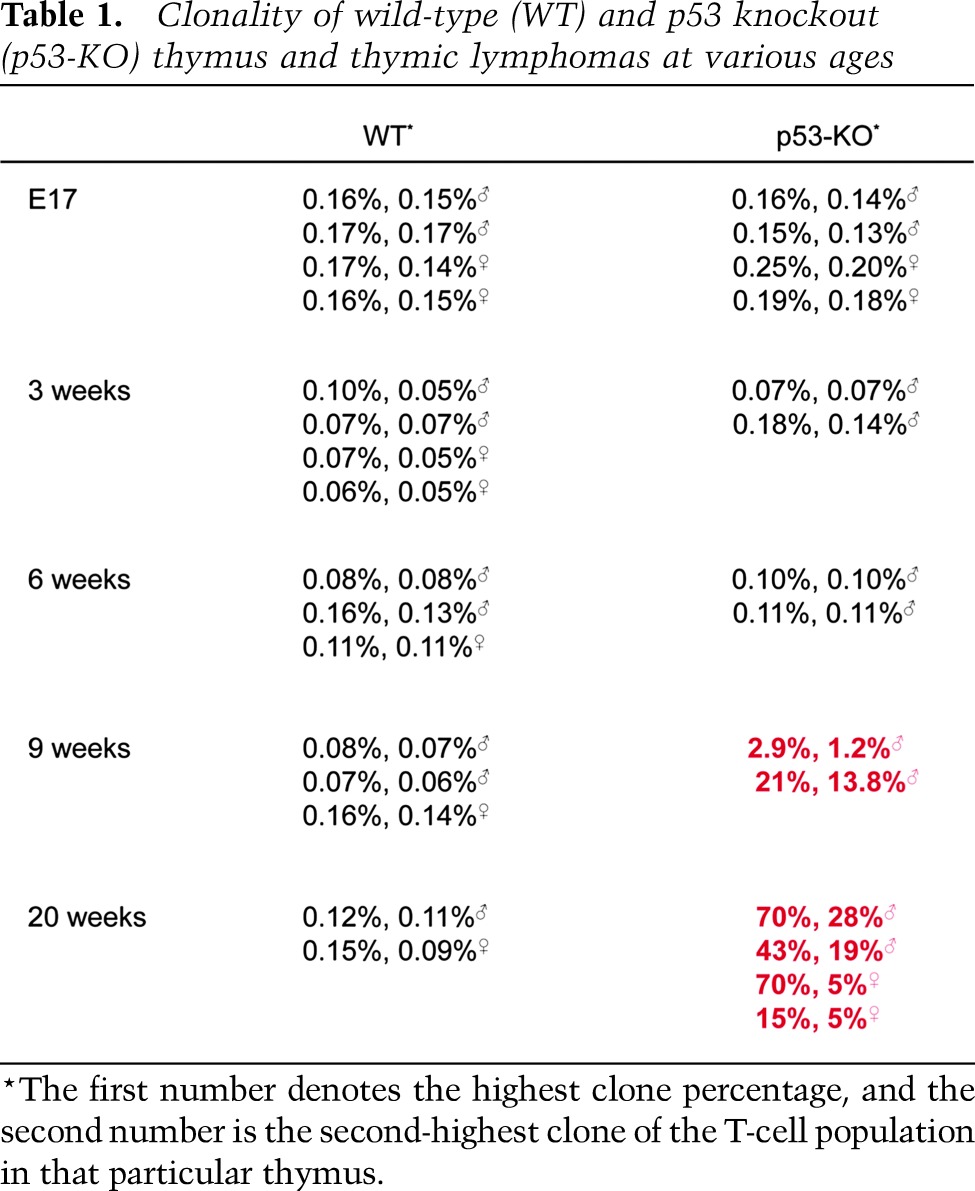
Clonality of wild-type (WT) and p53 knockout (p53-KO) thymus and thymic lymphomas at various ages

The increase in the frequency of replicated clones by 9 wk (30-fold to 200-fold) and 20 wk (120-fold to 600-fold) was a statistically significant difference from wild-type mice, and we employed this as the definition for determining that a thymic lymphoma had been formed and detected. The clonal expansion of these T cells is anomalous because they have not yet been exposed to antigen, so this could not be antigen-driven clonal expansion. Also, the loss of p53 itself did not cause a shift in the ratio of productive to nonproductive TCRβ sequences, as they remain similar in the 6-wk-old thymus (Supplemental Table 1). The ratio is reduced fourfold to fivefold in the p53 knockout thymic lymphomas, leading us to believe that another protein may be responsible for correct TCRβ selection.

**Figure 2.**
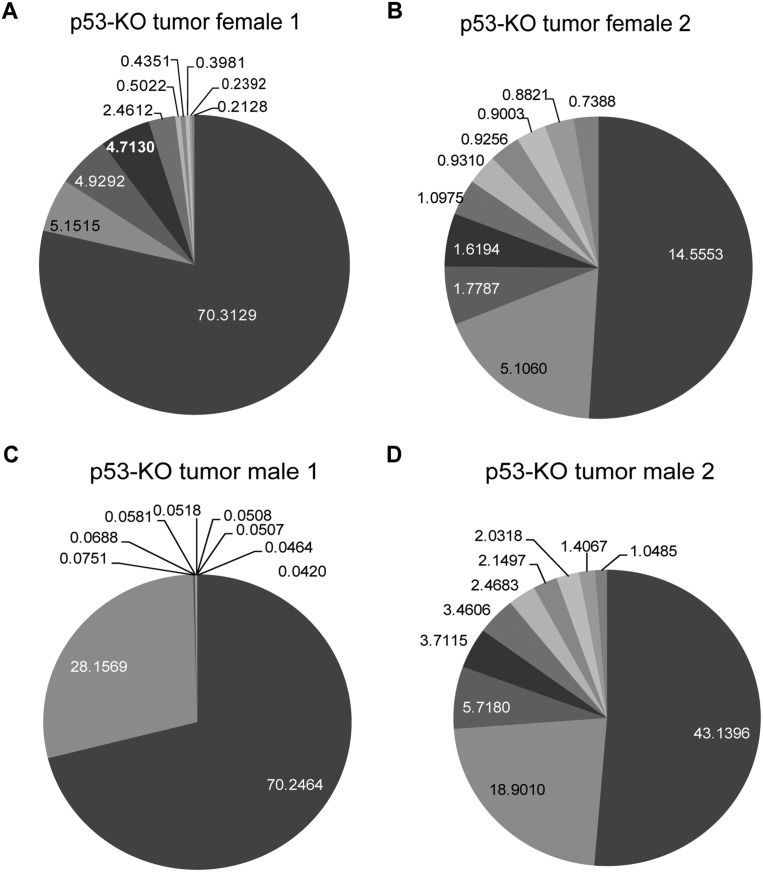
Oligoclonal p53 knockout (p53-KO) thymic lymphomas are comprised of a few dominant clones. (*A–D*) The top 10 clones for each thymic lymphoma were plotted in a pie chart to show dominant clones. The percentages for all clones can be seen at http://sns.ias.edu/∼cschan/TLymphoma.

The number of different clones (distinct TCR-β chains) contributing to the thymic lymphoma in a single p53 knockout mouse demonstrated that these tumors arose multiple independent times and were oligoclonal. By 20 wk, dominant clones arose (Fig. 2) so that, in one case, 98% of the T cells in the thymus arose from two different clones. In other cases, as many as 10 different clones contributed to these tumors.

The clonal frequencies for the total population of E17, 3 wk, 6 wk, 9 wk, 20 wk wild-type and p53 knockout thymic T cells, including the p53 knockout thymic lymphomas at 20 wk, are available at http://sns.ias.edu/∼cschan/TLymphoma.

### Whole-exome sequencing of p53 knockout thymic lymphomas

Having established the timing of tumorigenesis in the p53 knockout thymic lymphoma model, we can now ascertain potential driving factors for these transformations. To explore this, whole-exome sequencing of three independent p53 knockout thymic lymphoma samples obtained at 20 wk after birth was carried out. As a control sample of normal genomic DNA, matched tail DNA from a female C57Bl/6 mouse was employed. The nonsynonymous point mutations or stop codon/frameshift mutations detected by this sequencing did not identify any tumor driver mutations in similar genes between the three tumor DNA samples that were sequenced (Supplemental Table 2). Exon sequencing data are available at http://sns.ias.edu/∼cschan/TLymphoma.

### Copy number variations (CNVs) in p53 knockout thymic lymphomas

**Figure 3.**
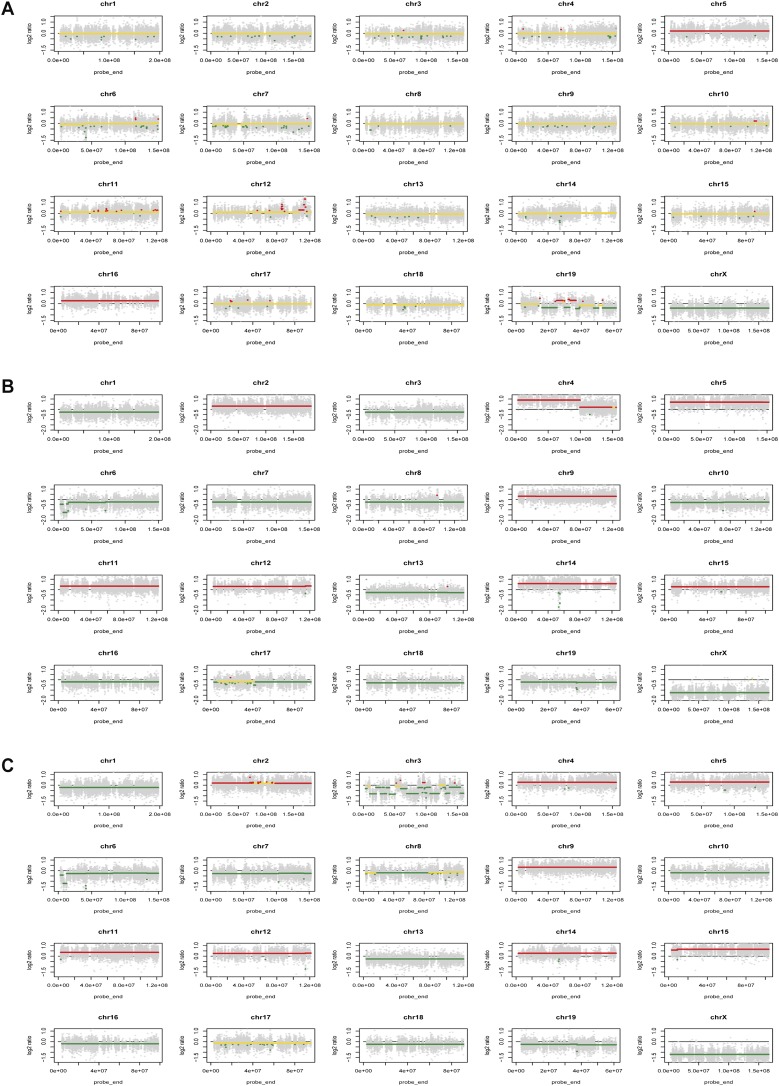
Increased CNVs in p53 knockout (p53-KO) thymic lymphomas. CNV analysis was completed as described in the Materials and Methods. CNV plots for female 1 p53 knockout thymic lymphoma (*A*), male 1 p53 knockout thymic lymphoma (*B*), and male 2 p53 knockout thymic lymphoma (*C*) are shown. Red lines indicate areas of amplification, yellow lines indicate no change, and green lines are areas of deletion.

**Figure 4.**
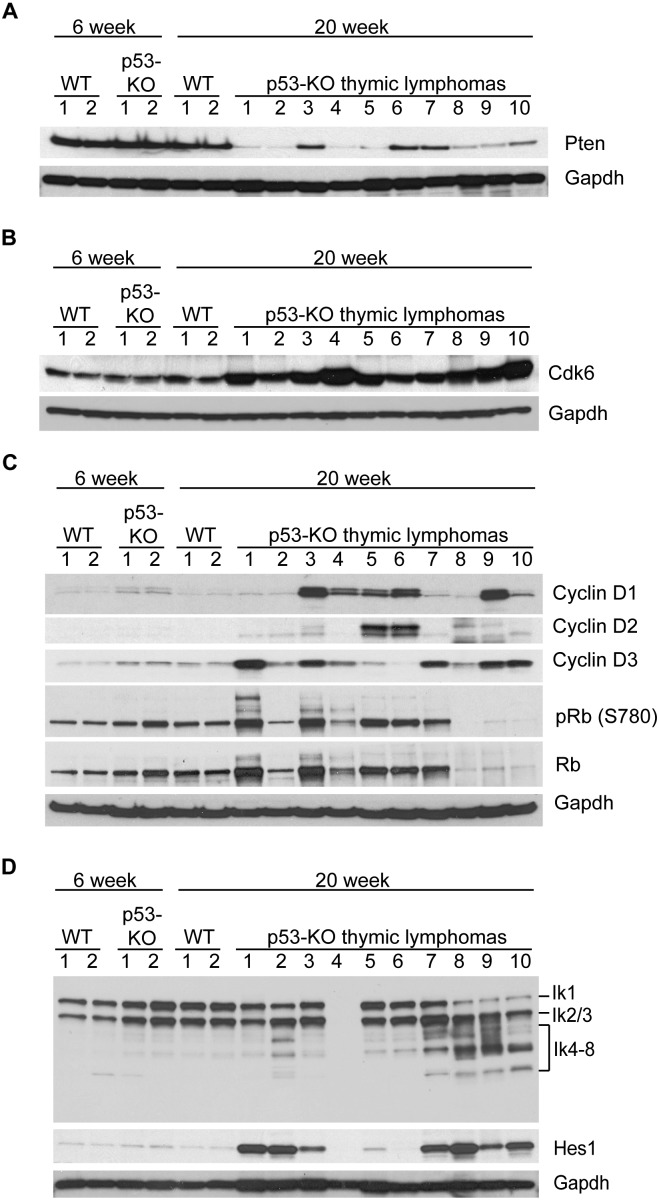
Pten loss, Cdk6 overexpression, and dominant-negative expression in p53 knockout (p53-KO) thymic lymphomas. Western blot of 6-wk-old wild-type (WT) and p53 knockout thymus and 20-wk-old wild-type and p53 knockout thymic lymphoma protein indicating the expression of Pten (*A*); Cdk6 (*B*); Cyclin D1, Cyclin D2, Cyclin D3, phospho-Rb, and Rb (*C*); and Ikaros isoforms (Ik1–8) and Hes1 (*D*), with Gapdh as a loading control (*A–D*).

Using the exome sequencing data, CNVs occurring within each of the three tumors sequenced were determined. Using a cutoff of −0.3 for deletions (corresponding to 38% of the tumor sample having deletion of one copy) and 0.2 for amplification (corresponding to 30% of the tumor sample having amplification of one copy), there are 276, 348, and 422 CNVs in the three tumors evaluated (Fig. 3). In contrast to the point mutations, these CNVs provided reproducible patterns of losses or gains of DNA copies. Of these, identical biallelic *Pten* deletions were found in every oligoclonal tumor within an individual mouse. However, individual p53 knockout mice had a different *Pten* deletion found in each of their oligoclonal tumors. The tumor of a male mouse with thymic lymphomas at 20 wk was composed of clones representing 39.4%, 13.5%, 8.4%, and 7.0% of the total tumor load, but the identical *Pten* deletion was represented by 85% of the DNA reads. In a second male mouse, the tumor was composed of two major clones at 76.7% and 9.7%, and 98% of the DNA reads had the same *Pten* deletion. These data are consistent with the idea that in each p53 knockout mouse, the *Pten* deletion occurred and was selected for future tumors during development prior to the creation of the TCRβ rearrangement and expression of the protein on the T-cell surface. In 10 tumors analyzed for Pten protein by Western blot, Pten deletion was found in seven, with three tumors having reduced levels of Pten (Fig. 4A). Other recurrent deletions were observed at lower frequencies in tumors and are listed in Supplemental Table 3. Amplifications and chromosomal aneuploidy were more difficult to interpret because there was no single gene or gene cluster with a significantly high copy number. Many of the amplification regions encompassed the entire chromosome (Fig. 3), which include chromosomes 2, 5, 9, 11, 12, 14, 15, and 16. Of these, only chromosome 5 was amplified in all three of the tumor samples. Taken together, there were 175 genes that are recurrently amplified in all tumors (Supplemental Table 4). These amplifications occurred in a smaller percentage of clones, unlike the Pten deletion, suggesting that these driver amplifications followed after TCRβ rearrangement. A comparison of this list with known human cancer driver genes revealed *Cdk6* and *Ikzf1* (Ikaros) as two genes whose amplifications may drive tumorigenesis.

### Pten, Cyclin D, Cdk6, and Ikaros expression in p53 knockout thymic lymphomas

Western blotting confirmed the loss or reduction of Pten expression in all p53 knockout thymic lymphomas samples when compared with normal thymus tissue or thymic protein from p53 knockout mice at 6 wk after birth, when lymphoma cells are not yet detectable and normal cells far exceed malignant cells (Fig. 4A). The Western blot employing antibodies to Cdk6 demonstrates that this gene is amplified and overexpressed in all 10 tumor samples compared with normal thymic T cells (Fig. 4B). Since Cdk6 requires the binding of Cyclin D to be fully functional, the levels of Cyclin D1, Cyclin D2, and Cyclin D3 in the tumors were determined. A very high expression of at least one type of Cyclin D for each tumor along with inactivation or loss of Rb in three tumors were observed (Fig. 4C). The Western blot employing antibodies against Ikaros, a transcription factor required for differentiation of thymic T cells, demonstrated dominant-negative isoforms of Ikaros (IK4–8) in seven of the tumors. Ikaros was found to be deleted in one tumor (Fig. 4D). The levels of the Hes1 protein (Kathrein et al. 2008; Kleinmann et al. 2008) were overexpressed in seven of the tumors, suggesting a pseudoactivation of the Notch1 pathway due to overexpression of Ik4–8 (Fig. 4D). Analysis of these same proteins in thymic lymphomas from the p53 mutant (R172H/R172H) revealed a similar expression profile (Supplemental Fig. 1). Taken together, these data support a role for the loss of p53 (genomic instability), the loss of Pten (altered metabolic regulation), the overexpression of Cyclin D–Cdk6 (cell cycle regulation), and altered splicing patterns of Ikaros (altering T-cell developmental pathways) in driving cellular proliferation and altering development during mouse T-cell lymphomagenesis.

## Discussion

Since all exonic mutations were sequenced in the tumors, we were able to estimate a mutation frequency for the p53 knockout thymic lymphoma. An average for the three tumors sequenced produced ∼100 point mutations found per 100 Mb of genomic DNA, giving a somatic point mutation frequency of one mutation per megabase of DNA. This somatic mutation frequency is similar to the mutational frequency observed with human breast cancers and pancreatic cancers (Lawrence et al. 2013). On the other hand, the frequency of CNVs detected in these tumors is very high, between 276 and 422 per genome for the three tumors. Two of the tumors have CNV patterns resembling chromothripsis, a pattern observed in human tumors with p53 mutations. The number of distinct clones of T cells contributing to a thymic lymphoma in a mouse between 9 wk and 20 wk after birth was ∼0.13–0.8 clones produced per day, reflecting the high frequency of CNVs observed. It is very likely that these numbers represent minimal estimates per day due to large variations caused naturally between mice and over time because of selective pressures for the most robust clones. While some mutations were common to most of these clones (*Pten* deletion, Cyclin D–Cdk6 overexpression, and Ikaros splicing changes), other mutations arising later in tumor evolution (by 20 wk after birth) produced dominant clones of T cells contributing to these lymphomas at later times. The high frequency of genomic instability (CNVs) driven by the inherited p53 mutations demonstrates the importance of p53 to the genetic integrity of somatic T cells.

Starting with a germline deletion of the p53 gene, the great majority of thymic lymphomas that are produced are a result of mutations in the same genes, which appear to be selected for in the same order of temporal events. Following the inherited p53 mutation, a Pten mutation is rapidly selected for prior to the development of mature T cells with receptors, which may also explain the decrease in the ratio of productive to nonproductive TCRβ sequences (Newton and Turka 2012). While the high mutation frequency in the absence of p53 gives rise to many types of mutations, the loss of Pten metabolically permits the selection of those cells during development in the T-cell lineage (and possibly other lineages) because of its role in the insulin-like growth factor pathway. This selective advantage would increase activated Akt, glucose uptake, and metabolic precursors needed for cell division. Then, after the T-cell receptor is added to the surface of T cells, additional mutations arise, such as the dominant-negative isoforms of IK4–8, which prevents terminal differentiation in this lineage. This might well then be followed by the overexpression of Cyclin D1, Cyclin D2, and Cyclin D3, which are clearly observed after the T-cell receptor is present, as is the Cdk6 amplification or Rb deletion. While Pten deletion and Cdk6 overexpression are found in all tumors, the choice of which D cyclin is overproduced and whether Ikaros dominant-negative isoforms are made occurs in some but not all tumors. These observations suggest that the presence of a germline p53 mutation sets an order to the selection of possible genes that contribute to the tumor not only when dominant clones arise but, in some cases, like Pten, for all clones that contribute to the tumor.

The genes that we identified are well known to drive T-cell leukemogenesis in mice as well as in humans. PTEN, CDK6, CCNDs, RB, and dominant-negative Ikaros isoform expression has been previously shown to be altered in human T-cell leukemias/lymphomas (Chilosi et al. 1998; Sun et al. 1999; Hatta and Koeffler 2002; Sicinska et al. 2003; Maser et al. 2007; Li et al. 2008; Gutierrez et al. 2009). Although we did not identify activating Notch1 mutations in the p53 knockout lymphoma that are common in T-ALL (T-cell adult lymphoblastic leukemia) (Weng et al. 2004; O’Neil et al. 2006), aberrant expression of Ikaros dominant-negative isoforms seems to substitute. Also, while p53 is normally wild type in T-ALL, Arf is biallelically deleted (Ferrando et al. 2002; Mullighan et al. 2008). However, by starting with a loss of p53 in our model, it appears that we removed the selective pressure for Arf deletion. Therefore, in this case, a p53 mutation functions as a gatekeeper mutation that sets a path of subsequent mutations and selection events leading rapidly and with a high frequency to oligoclonal tumors, providing the tumor heterogeneity that finally selects dominant clones. We will need to understand both the path chosen and the nature of the subsequent mutations that produce dominant clones. This model system provides that opportunity.

## Materials and methods

### Mice

All animal protocols used in this study were approved by the Rutgers Biomedical and Health Sciences Animal Care and Use Committee. C57BL/6 wild-type and p53 knockout mice were housed in a room maintained at 75°F and 50% humidity with 12-h light/dark cycles in microisolator cages and allowed access to water and chow ad libitum. For extraction of thymi, wild-type and p53 knockout mice were euthanized at the indicated times using CO_2_, and tissue was placed in liquid nitrogen for further analysis.

### DNA extraction and TCRβ sequencing

DNA was extracted from 30 mg of thymic tissue using the Purelink genomic DNA extraction (Life Technologies). The TCRβ locus was sequenced using the ImmunoSeq survey level assay by Adaptive Biotechologies with 500 ng of genomic DNA from wild-type and p53 knockout mice at various ages, including p53 knockout thymic lymphomas. TCRβ sequencing was analyzed using the ImmunoSeq Analyzer (Adaptive Biotechnologies).

### Exome sequencing and somatic point mutation analysis

Whole-exome sequencing and SNP/indel calling were completed by BGI Americas, Inc., using 3 μg of genomic DNA from p53 knockout thymic lymphomas female 1, male 1, and male 2. The tail DNA from female 1, taken at 3 wk of age, was used as a matched normal for all tumors. The exonic regions were captured using Agilent SureSelect Mouse All Exon 50 Mb and sequenced by 100-base-pair (bp) paired-end reads on an Illumina platform. The mm9 mouse reference genome was used to align good quality reads using Burrows-Wheeler alignment (BWA). The genome analysis toolkit (GATK) best practices workflow was followed to preprocess data. Sorting, merging, and indexing of the sequencing reads were accomplished with SAMtools 0.1.16. Picard 1.100 was used to mark PCR duplicates. To correct the misalignment caused by indels, local realignment was accomplished using RealignerTargetCreator and IndelRealigner of GATK (McKenna et al. 2010). The exome interval list (provided by the Agilent SureSelect Mouse All Exon kit) was used to identify the position of each exon in realignment. Recalibration of the base quality score was completed using GATK BaseRecalibrator. Somatic point mutations were called on matched normal and tumor BAM files using MuTect (Cibulskis et al. 2013). After identifying somatic genomic variants, known SNPs were identified by comparing with dbSNP128 for mice. After filtering known SNPs, somatic mutations were found in tumor samples as compared with normal samples.

### Structural variation analysis

CNV analysis was accomplished using ExomeCNV (Sathirapongsasuti et al. 2011) using the default parameter setting with the sample admixture rate set to 0.5. Briefly, DepthOfCoverage in GATK was used to convert BAM files (after removing PCR duplicates) into coverage files. The logarithm of coverage ratio between tumor and normal samples was calculated by ExomeCNV. ExomeCNV used this log ratio of read depth at each exon to call exonic CNVs and combine them into segments using the circular binary segmentation (CBS) algorithm (Olshen et al. 2004). Chromosome Y was not specified in our CNV analysis because we used the same female normal sample matched for both female and male tumor samples.

### Western blotting

Ten milligrams of tissue from thymus or thymic lymphomas was homogenized in 500 μL of 2× Laemmli buffer (0.125M Tris-HCl at pH 6.8, 10% β-mercaptoethanol, 4% sodium dodecyl sulphate, 20% glycerol, 0.05% bromophenol blue). Ten microliters of protein extract was subjected to SDS-PAGE and transferred to an Immobilon-P membrane (EMD Millipore). Western blot was carried out using antibodies to Pten (#9188), Cdk6 (#3136), Cyclin D1 (#2978), Cyclin D2 (#3741), Cyclin D3 (#2936), phospho-Rb (S780) (#9307), Rb (#9313), Ikaros (#5443), and Hes1 (#11988) (Cell Signaling) and Gapdh (#25778) (Santa Cruz Biotechnology, Inc.).

### Competing interest statement

The authors disclose no potential conflicts of interest.

## Supplementary Material

Supplemental Material
