# Supplementary material for: The evolution of thymic lymphomas in p53 knockout mice
Source: Genes Dev. 2014 Dec 1;28(23):2613–20. doi: 10.1101/gad.252148.114 (PMC4248292; doi:10.1101/gad.252148.114)
Supplement: Supplemental Material [file supp_28_23_2613__index.html]

Supplemental Material 

# The evolution of thymic lymphomas in p53 knockout mice

## Supplemental Material

**Files in this Data Supplement:**

- Supp Figure 1 Legends.docx
- Supp Figure 1.tif
- Supp Table 1.docx
- Supp Table 2.docx
- Supp Table 3.docx
- Supp Table 4.docx
