# Supplementary material for: The evolution of thymic lymphomas in p53 knockout mice
Source: Genes Dev. 2014 Dec 1;28(23):2613–20. doi: 10.1101/gad.252148.114 (PMC4248292; doi:10.1101/gad.252148.114)
Supplement: Supplemental Material [file supp_28.23.2613_Supp_Figure_1_Legend.docx]

**Supplemental Figure 1. Pten loss, Cdk6 overexpression, and dominant-negative Ikaros expression in p53 mutant thymic lymphomas.** Western blot of 20 week-old WT and p53 mutant (R172H/R172H) thymic lymphoma protein indicating the expression of Pten, Cdk6, Cyclins D1, D2, and D3, phospho-Rb, Rb, Ikaros isoforms (Ik1-8) and Hes1, with Gapdh as a loading control.
