# Supplementary figures and images for: The evolution of thymic lymphomas in p53 knockout mice
Source: Genes Dev. 2014 Dec 1;28(23):2613–20. doi: 10.1101/gad.252148.114 (PMC4248292; doi:10.1101/gad.252148.114)

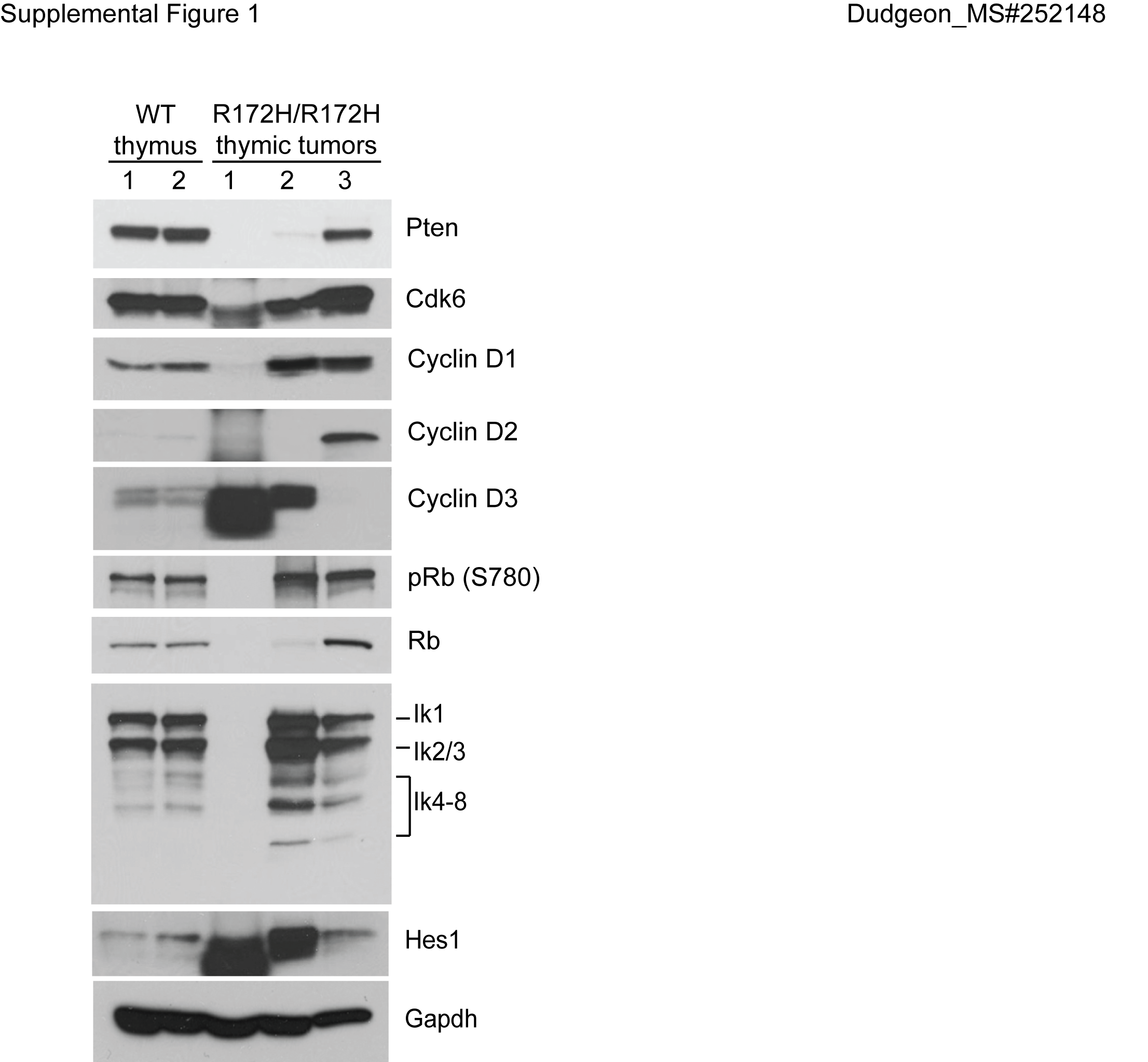

Supplement: Supplemental Material [file supp_28.23.2613_Supp_Figure_1.tif]
