# Supplementary material for: The evolution of thymic lymphomas in p53 knockout mice
Source: Genes Dev. 2014 Dec 1;28(23):2613–20. doi: 10.1101/gad.252148.114 (PMC4248292; doi:10.1101/gad.252148.114)
Supplement: Supplemental Material [file supp_28.23.2613_Supp_Table_1.docx]

| Age | Mouse | WT | p53-KO |  |
| --- | --- | --- | --- | --- |
| 6 weeks  20 weeks | male 1  male 2  female 1  male 1  male 2  female 1  female 2 | 2.80  2.61  2.75  2.63  2.74 | 2.75  2.70  0.48  0.55  0.60  0.50 | |

The number of unique sequences for WT samples is much greater than that of the 20 week p53-KO lymphoma samples. To maintain equality in calculating ratios based on the number of sequences analyzed, only the top 5000 highest frequency sequence were used (which is approximately the total number of unique sequences for lymphomas).

Supplemental Table 1. Ratio of Productive to Non-Productive TCRβ Sequences
