# Supplementary material for: The evolution of thymic lymphomas in p53 knockout mice
Source: Genes Dev. 2014 Dec 1;28(23):2613–20. doi: 10.1101/gad.252148.114 (PMC4248292; doi:10.1101/gad.252148.114)
Supplement: Supplemental Material [file supp_28.23.2613_Supp_Table_2.docx]

| **Tumor** | **Gene** | **Assession Number** | **Exon** | **cDNA Mutation** | **Protein Mutation** | **Chromosome** | **Mutation** |
| --- | --- | --- | --- | --- | --- | --- | --- |
| **female 1** | *Fry* | NM_172887 | exon 36 | C4699G | R1567G | chr 5 | C→G |
| Supplemental Table 2. Non-Synonymous and Stop-Gain Mutations in p53-KO Thymic Lymphomas. | *Zfp467* | NM_020589 | exon 5 | T1352G | V451G | chr 6 | A→C |
|  | *Vmn2r51* | NM_001105179 | exon 2 | T409C | C137R | chr 7 | A→G |
|  | *Olfr618* | NM_147047 | exon 1 | A25G | N9D | chr 7 | A→G |
|  | *F11* | NM_028066 | exon 14 | C1706T | T569M | chr 8 | G→A |
|  | *Mast3* | NM_199308 | exon 23 | G3001A | D1001N | chr 8 | C→T |
|  | *Olfr44* | NM_146830 | exon 2 | G64A | E22K | chr 9 | C→T |
|  | *Lman1l* | NM_199222 | exon 13 | C1396A | L466M | chr 9 | G→T |
|  | *Anxa2* | NM_007585 | exon 6 | G373C | E125Q | chr 9 | G→C |
|  | *Hhatl* | NM_029095 | exon 5 | G325A | A109T | chr 9 | C→T |
|  | *Olfr799* | NM_146927 | exon 1 | T236C | L79P | chr 10 | T→C |
|  | *Dnah17* | NM_001167746 | exon 26 | C4032G | S1344R | chr 11 | G→C |
|  | *Six4* | NM_011382 | exon 3 | A2204G | D735G | chr 12 | T→C |
|  | *3632451O06Rik* | NM_026142 | exon 2 | T1261C | F421L | chr 14 | A→G |
|  | *Srfbp1* | NM_026040 | exon 3 | T176C | L59S | chr 18 | T→C |
|  | *Cep192* | NM_027556 | exon 41 | C6910T | R2304X | chr 18 | C→T |
|  |  |  |  |  |  |  |  |
| **male 1** | *2010300C02Rik* | NM_028096 | exon 7 | C1588T | P530S | chr 1 | G→A |
|  | *Henmt1* | NM_001078646 | exon 3 | C89A | P30Q | chr 3 | C→A |
|  | *Unc13b* | NM_001081413 | exon 9 | A746G | N249S | chr 4 | A→G |
|  | *Gm13251* | NM_001085522 | exon 4 | C1696T | H566Y | chr 4 | G→A |
|  | *Aass* | NM_013930 | exon 2 | G97A | V33M | chr 6 | C→T |
|  | *Acsm4* | NM_178414 | exon 8 | G1087T | G363W | chr 7 | G→T |
|  | *Apobr* | NM_138310 | exon 2 | C2057A | T686K | chr 7 | C→A |
|  | *Kcnk1* | NM_008430 | exon 2 | G389T | G130V | chr 8 | G→T |
|  | *Lpcat2* | NM_173014 | exon 12 | G1291T | E431X | chr 8 | G→T |
|  | *Clasp2* | NM_029633 | exon 20 | T1921C | Y641H | chr 9 | T→C |
|  | *Madcam1* | NM_013591 | exon 4 | T886C | S296P | chr 10 | T→C |
|  | *Crhr1* | NM_007762 | exon 11 | A1064G | Q355R | chr 11 | A→G |
|  | *Zswim8* | NM_027996 | exon 12 | C2573T | A858V | chr 14 | C→T |
|  | *4930474N05Rik* | NM_175008 | exon 3 | C374G | S125C | chr 14 | C→G |
|  | *Cyp2d11* | NM_001104531 | exon 3 | T495A | D165E | chr 15 | A→T |
|  | *Pten* | NM_008960 | exon 3 | A184T | K62X | chr 19 | A→T |
|  |  |  |  |  |  |  |  |
| **male2** | *Dusp27* | NM_001160049 | exon 6 | G2689A | A897T | chr 1 | C→T |
|  | *Gnai1* | NM_010305 | exon 8 | C896T | A299V | chr 5 | G→A |
|  | *Nos1* | NM_008712 | exon 17 | C2758A | L920I | chr 5 | C→A |
|  | *Aass* | NM_013930 | exon 2 | G97A | V33M | chr 6 | C→T |
|  | *Prokr1* | NM_021381 | exon 3 | G1114A | A372T | chr 6 | C→T |
|  | *Tshz3* | NM_172298 | exon 2 | C1244T | T415I | chr 7 | C→T |
|  | *Tnpo2* | NM_145390 | exon 20 | T2252G | L751R | chr 8 | T→G |
|  | *Olfr857* | NM_001012265 | exon 1 | A673C | I225L | chr 9 | A→C |
|  | *Keap1* | NM_001110307 | exon 2 | C80G | A27G | chr 9 | G→C |
|  | *Bves* | NM_024285 | exon 5 | C584T | P195L | chr 10 | C→T |
|  | *Papolg* | NM_172555 | exon 10 | T893C | V298A | chr 11 | A→G |
|  | *Ctns* | NM_031251 | exon 9 | G819T | K273N | chr 11 | C→A |
|  | *Olfr20* | NM_146923 | exon 2 | T542C | M181T | chr 11 | T→C |
|  | *Ccdc57* | NM_027745 | exon 5 | G658A | A220T | chr 11 | C→T |
|  | *Sstr1* | NM_009216 | exon 2 | A592G | T198A | chr 12 | A→G |
|  | *Rmi1* | NM_028904 | exon 3 | G509A | R170H | chr 13 | G→A |
|  |  |  | exon 3 | A1231G | K411E |  | A→G |
|  |  |  | exon 3 | T1716G | C572W |  | T→G |
|  | *Sh3bp1* | NM_009164 | exon 13 | C1150T | L384F | chr 15 | C→T |
|  | *Tcp11* | NM_013687 | exon 8 | C1147T | R383W | chr 17 | G→A |
|  | *Sapcd1* | NM_023893 | exon 2 | G199C | A67P | chr 17 | C→G |

Exome sequencing data were analyzed using MuTect (http://www.broadinstitute.org/cancer/cga/mutect) to identify somatic point mutations of three p53-KO thymic lymphomas. A matched normal tail genomic sample from female 1 was used as a control. Underlined genes denote stop-gain mutations.
