# Supplementary material for: The evolution of thymic lymphomas in p53 knockout mice
Source: Genes Dev. 2014 Dec 1;28(23):2613–20. doi: 10.1101/gad.252148.114 (PMC4248292; doi:10.1101/gad.252148.114)
Supplement: Supplemental Material [file supp_28.23.2613_Supp_Table_3.docx]

| gene | female1 | male1 | male2 | chr | start (bp) | end (bp) |
| --- | --- | --- | --- | --- | --- | --- |
| *Gm10334* | -0.99 | -5.82 | -4.49 | 6 | 41252270 | 41475078 |
| *Gm5409* | -0.99 | -5.41 | -4.37 | 6 | 41252270 | 41475078 |
| *Gm5771* | -0.99 | -6.02 | -4.85 | 6 | 41252270 | 41475078 |
| *Prss1* | -0.99 | -5.49 | -4.47 | 6 | 41252270 | 41475078 |
| *Prss2* | -0.99 | -5.46 | -4.36 | 6 | 41252270 | 41475078 |
| *Prss3* | -0.99 | -5.27 | -4.30 | 6 | 41252270 | 41475078 |
| *Try10* | -0.99 | -4.98 | -4.30 | 6 | 41252270 | 41475078 |
| *Try4* | -0.99 | -5.34 | -4.74 | 6 | 41252270 | 41475078 |
| *Try5* | -0.99 | -6.12 | -4.57 | 6 | 41252270 | 41475078 |
| *Tfap2a* | -0.47 | -0.45 | -0.55 | 13 | 40811043 | 40829192 |
| *Irx1* | -0.41 | -0.64 | -0.61 | 13 | 72095679 | 72101171 |
| *Foxd1* | -0.51 | -0.42 | -0.51 | 13 | 99124199 | 99126660 |
| *A430107P09Rik* | -0.42 | -5.41 | -4.27 | 14 | 54285614 | 54288010 |
| *Pten* | -1.19 | -0.46 | -1.81 | 19 | 32832066 | 32900650 |
| *Ide* | -0.44 | -0.74 | -0.74 | 19 | 36806220 | 38480097 |
| *Rbp4* | -0.44 | -0.44 | -0.40 | 19 | 36806220 | 38480097 |

Supplemental Table 3. Recurrent Deleted Genes in p53-KO Thymic Lymphomas.

The values for copy number variations are presented as the log coverage ratio (log R ratio, or LRR) representing the log_2_ of the ratio of normalized coverage of tumor DNA to normal tail DNA. There is a cutoff of -0.3 for deletions (corresponding to 38% of a sample having deletion of 1 copy). Genes are ordered based on chromosome number then location.
