# Supplementary material for: The evolution of thymic lymphomas in p53 knockout mice
Source: Genes Dev. 2014 Dec 1;28(23):2613–20. doi: 10.1101/gad.252148.114 (PMC4248292; doi:10.1101/gad.252148.114)
Supplement: Supplemental Material [file supp_28.23.2613_Supp_Table_4.docx]

| gene | female1 | male1 | male2 | chr. | start (bp) | end (bp) |
| --- | --- | --- | --- | --- | --- | --- |
| *Rnf223* | 0.86 | 0.32 | 0.35 | 4 | 155506278 | 155507528 |
| *Cdk6* | 0.35 | 0.91 | 0.44 | 5 | 3344311 | 3522225 |
| *Emid1* | 0.26 | 0.45 | 0.44 | 11 | 5006268 | 5052225 |
| *Ikzf1* | 0.27 | 0.45 | 0.44 | 11 | 11586215 | 11672929 |
| *Grb10* | 0.27 | 0.45 | 0.44 | 11 | 11830501 | 11937423 |
| *Fbxw11* | 0.30 | 0.45 | 0.44 | 11 | 32542554 | 32646814 |
| *Ublcp1* | 0.31 | 0.45 | 0.44 | 11 | 44268072 | 44284050 |
| *Canx* | 0.26 | 0.45 | 0.44 | 11 | 50107458 | 50139175 |
| *N4bp3* | 0.25 | 0.45 | 0.44 | 11 | 51456590 | 51471183 |
| *Slc22a4* | 0.30 | 0.45 | 0.44 | 11 | 53796627 | 53841592 |
| *Slc36a2* | 0.30 | 0.45 | 0.44 | 11 | 54971969 | 54998579 |
| *2810021J22Rik* | 0.35 | 0.45 | 0.44 | 11 | 58606303 | 58717727 |
| *Fam183b* | 0.35 | 0.45 | 0.44 | 11 | 58606303 | 58717727 |
| *Olfr311* | 0.35 | 0.45 | 0.44 | 11 | 58606303 | 58717727 |
| *Olfr312* | 0.35 | 0.45 | 0.44 | 11 | 58606303 | 58717727 |
| *Olfr313* | 0.35 | 0.45 | 0.44 | 11 | 58606303 | 58717727 |
| *Zfp39* | 0.35 | 0.45 | 0.44 | 11 | 58606303 | 58717727 |
| *2810408A11Rik* | 0.38 | 0.45  Supplemental Table 4. Recurrent Amplified Genes in p53-KO Thymic Lymphomas. | 0.44 | 11 | 69710859 | 69735460 |
| *Eif5a* | 0.38 | 0.45 | 0.44 | 11 | 69710859 | 69735460 |
| *Gps2* | 0.38 | 0.45 | 0.44 | 11 | 69710859 | 69735460 |
| *Neurl4* | 0.38 | 0.45 | 0.44 | 11 | 69710859 | 69735460 |
| *Ybx2* | 0.26 | 0.45 | 0.44 | 11 | 69749400 | 69755101 |
| *Acadvl* | 0.28 | 0.45 | 0.44 | 11 | 69823684 | 69828930 |
| *Alox12e* | 0.26 | 0.45 | 0.44 | 11 | 70129114 | 70136020 |
| *4930544D05Rik* | 0.27 | 0.45 | 0.44 | 11 | 70428384 | 70432696 |
| *Chrne* | 0.27 | 0.45 | 0.44 | 11 | 70428384 | 70432696 |
| *C1qbp* | 0.25 | 0.45 | 0.44 | 11 | 70783701 | 70796528 |
| *Rpain* | 0.25 | 0.45 | 0.44 | 11 | 70783701 | 70796528 |
| *P2rx1* | 0.30 | 0.45 | 0.44 | 11 | 72812646 | 72828699 |
| *Fam222b* | 0.30 | 0.45 | 0.44 | 11 | 77908174 | 77970841 |
| *Nlk* | 0.28 | 0.45 | 0.44 | 11 | 78380669 | 78510927 |
| *Nos2* | 0.25 | 0.45 | 0.44 | 11 | 78734360 | 78773626 |
| *Lgals9* | 0.34 | 0.45 | 0.44 | 11 | 78776480 | 78798426 |
| *Wsb1* | 0.27 | 0.45 | 0.44 | 11 | 79052883 | 79068197 |
| *Rab11fip4* | 0.27 | 0.45 | 0.44 | 11 | 79404713 | 79507514 |
| *Ap2b1* | 0.27 | 0.45 | 0.44 | 11 | 83116198 | 83218535 |
| *Bcas3* | 0.26 | 0.45 | 0.44 | 11 | 85166665 | 85645890 |
| *Med13* | 0.27 | 0.45 | 0.44 | 11 | 86079216 | 86171027 |
| *Rps6kb1* | 0.28 | 0.45 | 0.44 | 11 | 86312512 | 86358309 |
| *Dhx40* | 0.25 | 0.45 | 0.44 | 11 | 86582350 | 86621162 |
| *Hsf5* | 0.28 | 0.45 | 0.44 | 11 | 87430665 | 87473044 |
| *Rnf43* | 0.26 | 0.45 | 0.44 | 11 | 87476588 | 87549041 |
| *Ppp1r9b* | 0.26 | 0.45 | 0.44 | 11 | 94852525 | 94868212 |
| *Kat7* | 0.30 | 0.45 | 0.44 | 11 | 95133166 | 95171560 |
| *Fam117a* | 0.32 | 0.45 | 0.44 | 11 | 95198331 | 95243186 |
| *Prkar2b* | 0.34 | 0.41 | 0.33 | 12 | 32643343 | 32746144 |
| *Npas3* | 0.27 | 0.41 | 0.33 | 12 | 54349663 | 55173162 |
| *Srp54a* | 0.28 | 0.41 | 0.33 | 12 | 56181482 | 56216354 |
| *Rab15* | 0.27 | 0.41 | 0.33 | 12 | 77898949 | 77923511 |
| *Fntb* | 0.28 | 0.41 | 0.33 | 12 | 77938453 | 78022399 |
| *Plek2* | 0.29 | 0.41 | 0.33 | 12 | 79989683 | 80007925 |
| *Abcd4* | 0.44 | 0.41 | 0.33 | 12 | 85626245 | 86818219 |
| *Acyp1* | 0.44 | 0.41 | 0.33 | 12 | 85626245 | 86818219 |
| *Aldh6a1* | 0.44 | 0.41 | 0.33 | 12 | 85626245 | 86818219 |
| *Arel1* | 0.44 | 0.41 | 0.33 | 12 | 85626245 | 86818219 |
| *Ccdc176* | 0.44 | 0.41 | 0.33 | 12 | 85626245 | 86818219 |
| *Coq6* | 0.44 | 0.41 | 0.33 | 12 | 85626245 | 86818219 |
| *Dlst* | 0.44 | 0.41 | 0.33 | 12 | 85626245 | 86818219 |
| *Eif2b2* | 0.44 | 0.41 | 0.33 | 12 | 85626245 | 86818219 |
| *Entpd5* | 0.44 | 0.41 | 0.33 | 12 | 85626245 | 86818219 |
| *Fam161b* | 0.44 | 0.41 | 0.33 | 12 | 85626245 | 86818219 |
| *Fcf1* | 0.44 | 0.41 | 0.33 | 12 | 85626245 | 86818219 |
| *Fos* | 0.44 | 0.41 | 0.33 | 12 | 85626245 | 86818219 |
| *Isca2* | 0.44 | 0.41 | 0.33 | 12 | 85626245 | 86818219 |
| *Lin52* | 0.44 | 0.41 | 0.33 | 12 | 85626245 | 86818219 |
| *Ltbp2* | 0.44 | 0.41 | 0.33 | 12 | 85626245 | 86818219 |
| *Mlh3* | 0.44 | 0.41 | 0.33 | 12 | 85626245 | 86818219 |
| *Nek9* | 0.44 | 0.41 | 0.33 | 12 | 85626245 | 86818219 |
| *Npc2* | 0.44 | 0.41 | 0.33 | 12 | 85626245 | 86818219 |
| *Pgf* | 0.44 | 0.41 | 0.33 | 12 | 85626245 | 86818219 |
| *Prox2* | 0.44 | 0.41 | 0.33 | 12 | 85626245 | 86818219 |
| *Ptgr2* | 0.44 | 0.41 | 0.33 | 12 | 85626245 | 86818219 |
| *Rnf113a2* | 0.44 | 0.41 | 0.33 | 12 | 85626245 | 86818219 |
| *Rps6kl1* | 0.44 | 0.41 | 0.33 | 12 | 85626245 | 86818219 |
| *Syndig1l* | 0.44 | 0.41 | 0.33 | 12 | 85626245 | 86818219 |
| *Tmed10* | 0.44 | 0.41 | 0.33 | 12 | 85626245 | 86818219 |
| *Vrtn* | 0.44 | 0.41 | 0.33 | 12 | 85626245 | 86818219 |
| *Vsx2* | 0.44 | 0.41 | 0.33 | 12 | 85626245 | 86818219 |
| *Ylpm1* | 0.44 | 0.41 | 0.33 | 12 | 85626245 | 86818219 |
| *Zc2hc1c* | 0.44 | 0.41 | 0.33 | 12 | 85626245 | 86818219 |
| *Zfp410* | 0.44 | 0.41 | 0.33 | 12 | 85626245 | 86818219 |
| *Jdp2* | 0.60 | 0.41 | 0.33 | 12 | 86940054 | 86980828 |
| *Batf* | 0.28 | 0.41 | 0.33 | 12 | 87027669 | 87050037 |
| *Mfsd7c* | 0.26 | 0.41 | 0.33 | 12 | 87087488 | 87154535 |
| *Gpatch2l* | 0.31 | 0.41 | 0.33 | 12 | 87582827 | 87632318 |
| *Esrrb* | 0.26 | 0.41 | 0.33 | 12 | 87702066 | 87862578 |
| *Sptlc2* | 0.28 | 0.41 | 0.33 | 12 | 88648838 | 88729180 |
| *Nrde2* | 0.35 | 0.41 | 0.33 | 12 | 101363661 | 101397863 |
| *Rps6ka5* | 0.26 | 0.41 | 0.33 | 12 | 101787987 | 101963238 |
| *Golga5* | 0.25 | 0.41 | 0.33 | 12 | 103707343 | 103736117 |
| *Itpk1* | 0.35 | 0.41 | 0.33 | 12 | 103806792 | 103943079 |
| *Asb2* | 0.34 | 0.41 | 0.33 | 12 | 104559351 | 104594211 |
| *Otub2* | 0.38 | 0.41 | 0.33 | 12 | 104615103 | 104644560 |
| *Begain* | 0.28 | 0.41 | 0.33 | 12 | 109344640 | 110950646 |
| *Ccdc85c* | 0.28 | 0.41 | 0.33 | 12 | 109344640 | 110950646 |
| *Ccnk* | 0.28 | 0.41 | 0.33 | 12 | 109344640 | 110950646 |
| *Cyp46a1* | 0.28 | 0.41 | 0.33 | 12 | 109344640 | 110950646 |
| *Degs2* | 0.28 | 0.41 | 0.33 | 12 | 109344640 | 110950646 |
| *Dlk1* | 0.28 | 0.41 | 0.33 | 12 | 109344640 | 110950646 |
| *Eml1* | 0.28 | 0.41 | 0.33 | 12 | 109344640 | 110950646 |
| *Evl* | 0.28 | 0.41 | 0.33 | 12 | 109344640 | 110950646 |
| *Hhipl1* | 0.28 | 0.41 | 0.33 | 12 | 109344640 | 110950646 |
| *Rtl1* | 0.28 | 0.41 | 0.33 | 12 | 109344640 | 110950646 |
| *Setd3* | 0.28 | 0.41 | 0.33 | 12 | 109344640 | 110950646 |
| *Slc25a29* | 0.28 | 0.41 | 0.33 | 12 | 109344640 | 110950646 |
| *Slc25a47* | 0.28 | 0.41 | 0.33 | 12 | 109344640 | 110950646 |
| *Wars* | 0.28 | 0.41 | 0.33 | 12 | 109344640 | 110950646 |
| *Wdr25* | 0.28 | 0.41 | 0.33 | 12 | 109344640 | 110950646 |
| *Yy1* | 0.28 | 0.41 | 0.33 | 12 | 109344640 | 110950646 |
| *1700001K19Rik* | 0.41 | 0.45 | 0.35 | 12 | 110971980 | 112615929 |
| *Amn* | 0.41 | 0.45 | 0.35 | 12 | 110971980 | 112615929 |
| *Ankrd9* | 0.41 | 0.45 | 0.35 | 12 | 110971980 | 112615929 |
| *Cdc42bpb* | 0.41 | 0.45 | 0.35 | 12 | 110971980 | 112615929 |
| *Cinp* | 0.41 | 0.45 | 0.35 | 12 | 110971980 | 112615929 |
| *Dync1h1* | 0.41 | 0.45 | 0.35 | 12 | 110971980 | 112615929 |
| *Hsp90aa1* | 0.41 | 0.45 | 0.35 | 12 | 110971980 | 112615929 |
| *Rcor1* | 0.41 | 0.45 | 0.35 | 12 | 110971980 | 112615929 |
| *Stk30* | 0.41 | 0.45 | 0.35 | 12 | 110971980 | 112615929 |
| *Tecpr2* | 0.41 | 0.45 | 0.35 | 12 | 110971980 | 112615929 |
| *Traf3* | 0.41 | 0.45 | 0.35 | 12 | 110971980 | 112615929 |
| *Wdr20a* | 0.41 | 0.45 | 0.35 | 12 | 110971980 | 112615929 |
| *Zfp839* | 0.41 | 0.45 | 0.35 | 12 | 110971980 | 112615929 |
| *Ppp2r5c* | 0.41 | 0.43 | 0.35 | 12 | 110971980 | 112615929 |
| *2010107E04Rik* | 0.82 | 0.45 | 0.35 | 12 | 112645019 | 113419958 |
| *A230065H16Rik* | 0.82 | 0.45 | 0.35 | 12 | 112645019 | 113419958 |
| *Apopt1* | 0.82 | 0.45 | 0.35 | 12 | 112645019 | 113419958 |
| *Aspg* | 0.82 | 0.45 | 0.35 | 12 | 112645019 | 113419958 |
| *Bag5* | 0.82 | 0.45 | 0.35 | 12 | 112645019 | 113419958 |
| *BC048943* | 0.82 | 0.45 | 0.35 | 12 | 112645019 | 113419958 |
| *Ckb* | 0.82 | 0.45 | 0.35 | 12 | 112645019 | 113419958 |
| *Eif5* | 0.82 | 0.45 | 0.35 | 12 | 112645019 | 113419958 |
| *Exoc3l4* | 0.82 | 0.45 | 0.35 | 12 | 112645019 | 113419958 |
| *Gm266* | 0.82 | 0.45 | 0.35 | 12 | 112645019 | 113419958 |
| *Kif26a* | 0.82 | 0.45 | 0.35 | 12 | 112645019 | 113419958 |
| *Klc1* | 0.82 | 0.45 | 0.35 | 12 | 112645019 | 113419958 |
| *Mark3* | 0.82 | 0.45 | 0.35 | 12 | 112645019 | 113419958 |
| *Ppp1r13b* | 0.82 | 0.45 | 0.35 | 12 | 112645019 | 113419958 |
| *Tdrd9* | 0.82 | 0.45 | 0.35 | 12 | 112645019 | 113419958 |
| *Tnfaip2* | 0.82 | 0.45 | 0.35 | 12 | 112645019 | 113419958 |
| *Trmt61a* | 0.82 | 0.45 | 0.35 | 12 | 112645019 | 113419958 |
| *Xrcc3* | 0.82 | 0.45 | 0.35 | 12 | 112645019 | 113419958 |
| *Zfyve21* | 0.82 | 0.45 | 0.35 | 12 | 112645019 | 113419958 |
| *4930427A07Rik* | 1.32 | 0.45 | 0.35 | 12 | 113649233 | 114427733 |
| *A530016L24Rik* | 1.32 | 0.45 | 0.35 | 12 | 113649233 | 114427733 |
| *Adssl1* | 1.32 | 0.45 | 0.35 | 12 | 113649233 | 114427733 |
| *Akt1* | 1.32 | 0.45 | 0.35 | 12 | 113649233 | 114427733 |
| *BC022687* | 1.32 | 0.45 | 0.35 | 12 | 113649233 | 114427733 |
| *Brf1* | 1.32 | 0.45 | 0.35 | 12 | 113649233 | 114427733 |
| *Btbd6* | 1.32 | 0.45 | 0.35 | 12 | 113649233 | 114427733 |
| *Cdca4* | 1.32 | 0.45 | 0.35 | 12 | 113649233 | 114427733 |
| *Cep170b* | 1.32 | 0.45 | 0.35 | 12 | 113649233 | 114427733 |
| *Crip1* | 1.32 | 0.45 | 0.35 | 12 | 113649233 | 114427733 |
| *Crip2* | 1.32 | 0.45 | 0.35 | 12 | 113649233 | 114427733 |
| *Gpr132* | 1.32 | 0.45 | 0.35 | 12 | 113649233 | 114427733 |
| *Inf2* | 1.32 | 0.45 | 0.35 | 12 | 113649233 | 114427733 |
| *Jag2* | 1.32 | 0.45 | 0.35 | 12 | 113649233 | 114427733 |
| *Mta1* | 1.32 | 0.45 | 0.35 | 12 | 113649233 | 114427733 |
| *Nudt14* | 1.32 | 0.45 | 0.35 | 12 | 113649233 | 114427733 |
| *Pacs2* | 1.32 | 0.45 | 0.35 | 12 | 113649233 | 114427733 |
| *Pld4* | 1.32 | 0.45 | 0.35 | 12 | 113649233 | 114427733 |
| *Siva1* | 1.32 | 0.45 | 0.35 | 12 | 113649233 | 114427733 |
| *Tex22* | 1.32 | 0.45 | 0.35 | 12 | 113649233 | 114427733 |
| *Tmem121* | 1.32 | 0.45 | 0.35 | 12 | 113649233 | 114427733 |
| *Tmem179* | 1.32 | 0.45 | 0.35 | 12 | 113649233 | 114427733 |
| *Zbtb42* | 1.32 | 0.45 | 0.35 | 12 | 113649233 | 114427733 |
| *Adam6a* | 0.73 | 0.45 | 0.35 | 12 | 114727775 | 114784625 |
| *Adam6b* | 0.73 | 0.45 | 0.35 | 12 | 114727775 | 114784625 |
| *Wdr60* | 0.33 | 0.45 | 0.35 | 12 | 117445522 | 117501498 |
| *Esyt2* | 0.41 | 0.45 | 0.35 | 12 | 117519694 | 117611571 |
| *Ptprn2* | 0.39 | 0.45 | 0.35 | 12 | 117724192 | 118575485 |
| *Tmem196* | 0.30 | 0.49 | 0.34 | 12 | 121184434 | 121257179 |
| *Msra* | 0.27 | 0.67 | 0.35 | 14 | 64741457 | 65074740 |
| *Pebp4* | 0.27 | 0.67 | 0.35 | 14 | 70240226 | 70459693 |
| *Hs6st3* | 0.25 | 0.67 | 0.37 | 14 | 119537486 | 120269037 |
| *Enthd1* | 0.26 | 0.40 | 0.73 | 15 | 80282669 | 80390900 |

The values for copy number variations are presented as the log coverage ratio (log R ratio, or LRR) representing the log_2_ of the ratio of normalized coverage of tumor DNA to normal tail DNA. There is a cutoff of 0.2 for amplifications (corresponding to 30% of a sample having amplification of 1 copy). Genes are ordered based on chromosome number then location.
